# Role of Tocochromanols in Tolerance of Cereals to Biotic Stresses: Specific Focus on Pathogenic and Toxigenic Fungal Species

**DOI:** 10.3390/ijms23169303

**Published:** 2022-08-18

**Authors:** Jean-Marie Savignac, Vessela Atanasova, Sylvain Chéreau, Véronique Ortéga, Florence Richard-Forget

**Affiliations:** 1 INRAE, UR 1264 Mycology and Food Safety (MycSA), 33882 Villenave d’Ornon, France; 2 Syngenta France SAS, 32220 Lombez, France

**Keywords:** tocochromanol, tocopherol, tocotrienol, cereal, fungal pathogen, mycotoxin, plant defense

## Abstract

Fungal pathogens capable of producing mycotoxins are one of the main threats to the cultivation of cereals and the safety of the harvested kernels. Improving the resistance of crops to fungal disease and accumulation of mycotoxins is therefore a crucial issue. Achieving this goal requires a deep understanding of plant defense mechanisms, most of them involving specialized metabolites. However, while numerous studies have addressed the contribution of phenylpropanoids and carotenoids to plant chemical defense, very few have dealt with tocochromanols. Tocochromanols, which encompass tocopherols and tocotrienols and constitute the vitamin E family, are widely distributed in cereal kernels; their biosynthetic pathway has been extensively studied with the aim to enrich plant oils and combat vitamin E deficiency in humans. Here we provide strong assumptions arguing in favor of an involvement of tocochromanols in plant–fungal pathogen interactions. These assumptions are based on both direct effects resulting from their capacity to scavenge reactive oxygen species, including lipid peroxyl radicals, on their potential to inhibit fungal growth and mycotoxin yield, and on more indirect effects mainly based on their role in plant protection against abiotic stresses.

## 1. Introduction

For billions of people around the world, cereals provide the most accessible source of energy and are the principal components of the diet. Cereals rank among the most widespread crops worldwide. In 2020, world cereal production amounted to over 2650 Mt; this production is projected to expand by 375 Mt to reach more than 3000 Mt in 2029 [1]. Cereal production can, however, be injured by various types of stress, both abiotic and biotic, whose combined effect can adversely affect yield performance and grain quality. The main abiotic stresses that plants have to face include extreme temperatures, drought, salinity, and soil nutrient limitations. Regarding biotic stresses, viruses, fungi, bacteria, weeds, insects, and other pests can lead to more than 20% crop yield losses, as illustrated by the recent survey published by Savary et al. [2]. In addition to direct yield losses, some fungal species infecting cereals can also produce mycotoxins that are harmful to humans and livestock and pose potential health threats. The major mycotoxin-producing fungal genera are *Aspergillus* (producer of aflatoxins and ochratoxins), *Fusarium* (producer of trichothecenes, zearalenone, and fumonisins), and *Penicillium* (producer of ochratoxins). Whereas in northern countries, *Aspergillus* and *Penicillium* species are commonly considered as storage fungi since they usually invade grains during storage, *Fusarium* species most frequently invade the grains before harvest while the crop is still in the field. Actually, *Fusarium* head blight in small grain cereals and *Giberella* ear rot and *Fusarium* ear rot in maize, are all three caused by *Fusarium* species and are major devastating diseases that are extremely challenging to control.

To cope with such a diversity of biotic and abiotic stresses, plants have evolved multiple defense mechanisms, several of them relying on secondary (or specialized) metabolites and hormones [3]. Plant secondary metabolites are divided into three major groups: phenylpropanoids, terpenoids, and N- and S-containing compounds. Phenylpropanoids, both flavonoids and non-flavonoid compounds, have been the most widely studied for their contribution to plant defense mechanisms [4]. Phenylpropanoids have been proven to play a central role in plant defense through their capacity to minimize the generation of reactive oxygen species (ROS) and to quench ROS once they are formed and therefore protect plants from oxidative damage [5]. In addition, many phenylpropanoids have been shown to possess antibacterial and antifungal properties, to act as plant defense system mediators and, for some of them, as precursors of physical defense processes such as cell wall lignification [6]. Indeed, lignin and lignans that are responsible of cell wall thickening are nearly exclusively based on phenylpropanoid units derived from the oxidative polymerization of hydroxycinnamoyl alcohol derivatives [7]. Moreover, in addition to displaying antifungal properties, several phenylpropanoids can affect the production of mycotoxins. There is notably a large body of evidence that supports the inhibitory activities of hydroxycinnamic acid derivatives, mainly *p*-coumaric, ferulic, caffeic, and chlorogenic acids, towards the biosynthesis of various mycotoxins including deoxynivalenol, type A trichothecenes, fumonisins, and aflatoxins [8]. Some flavonoids (e.g., naringenin and quercetin) have also been shown to exert an inhibitory effect on mycotoxin production, such as type B trichothecenes [9]. In addition to phenylpropanoids, plant-specialized terpenoids are also considered as key chemical mediators of abiotic and biotic interactions [10]. Terpenoids are the largest class of secondary metabolites in plants, characterized by a high diversity of physical and chemical properties, including hydrophilic, lipophilic, and volatile or non-volatile metabolites. Much attention has been paid to the role of the volatile fraction of terpenoids, predominantly consisting of hemiterpenoids, monoterpenoids and sesquiterpenoids, in controlling pests [11]. Additionally, some volatile compounds, such as linalool derivatives, have been shown to be released by plant tissues in response to fungal infection and to act as antifungal agents [9]. An antifungal and antimicrobial activity has also been demonstrated for some non-volatile diterpenoids, such as phytocassane, which can disrupt microbial membranes [12]. In addition, there is a large body of evidence for an active and important role of abscisic acid, a sesquiterpenoid plant hormone, in plant-pathogen interactions. This role can be related to its regulatory effect on callose deposition, which provides physical barriers against plant fungal pathogens and to its capacity to control stomatal aperture [13]. Moreover, abscisic acid has been proven to interact with the ethylene signaling pathway and to counteract the spread of some necrotrophic fungal pathogens that exploit the ethylene signaling pathway to enhance colonization of plant tissues [14]. In addition to their role in the biosynthesis of abscisic acid, carotenoids, a group of tetraterpenoids, possess strong antioxidant capacities and are capable of quenching various ROS and, notably, the lipid peroxyl radical (LOO•), thus providing plant cell membrane protection [15]. As demonstrated for capsanthin and capsorubin [16], plant carotenoids are also likely to act as antifungal and antimycotoxin agents. Similarly, tocochromanols are plant compounds with a strong antioxidant potential. The biosynthesis of this class of compounds draws on metabolites from the terpenoid and shikimate pathways. Tocochromanols are acknowledged to efficiently quench singlet oxygen and scavenge various radicals, especially lipid peroxyl radicals derived from polyunsaturated fatty acids, thereby terminating lipid peroxidation chain reactions [17]. However, notwithstanding their potent antioxidant activity, their ecological role in plant defense mechanisms has been much less studied and mainly restricted to a contribution to resistance to abiotic stresses [18,19,20,21,22]. Regarding tolerance to light stress, the protective role of tocochromanols, which are located in photosynthetic membranes, has been ascribed to their capacity to scavenge singlet oxygen produced in the photosystem II triplet [23]. Their contribution to drought stress tolerance has been related to their capacity to affect the fluidity of thylakoids or to alter intracellular signaling by modulating jasmonic acid synthesis [18]. Such physiological roles of tocochromanols strongly argue in favor of a key contribution of this class of as yet scarcely studied compounds in plant–pathogen interactions.

The aim of this review is to gather the most recent knowledge on tocochromanols in cereals and to highlight recent insights that support the key role this class of compounds could play in the mechanisms cereals employ to cope with biotic stresses, notably with the threat represented by toxigenic fungal species.

## 2. Tocochromanols in Cereals

### 2.1. Tocochromanol Structure

**Figure 1:**
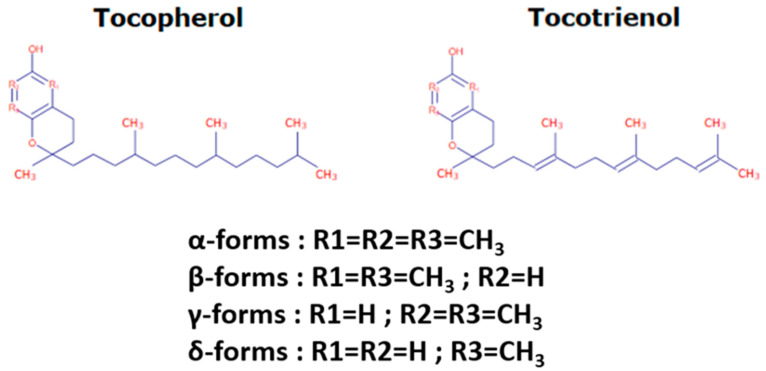
Tocochromanol structure.

Tocochromanols encompass tocopherols and tocotrienols and are the main components of the vitamin E family. They are exclusively synthesized by photosynthetic eukaryotes and other oxygenic photosynthetic organisms, such as cyanobacteria, and are essential phytonutrients for mammals. In addition, whereas tocopherols are widely distributed in higher plants, tocotrienols occur only in some non-photosynthetic tissues including seeds, roots, and tubers [24]. Their general structure (reported in Figure 1) consists of a polar chromanol ring and a hydrophobic 16-carbon side chain attached to the ring via the C-2 atom. While tocopherols have fully saturated 16-carbon phytol side-chains, tocotrienols contain geranylgeranyl side chains with three double bonds in positions C-3, C-7, and C-11. As indicated in Figure 1, both tocopherols and tocotrienols can occur as four isomers (α, β-, γ-, and δ-) that differ from each other by the number and position of methyl groups in the chromanol ring. The α-isomers are tri-methylated in positions 5, 7, and 8, β- and γ-tocochromanols are di-methylated in position 5 and 8 or in position 7 and 8, respectively, and δ-isomers are methylated in position 8. The chromanol ring forms the basis for the high antioxidant potency of tocochromanols, this potency being modulated by the structure and length of the isoprenoid chains. Actually, tocochromanols are acknowledged as the lipid-soluble antioxidant metabolites possessing the highest capacity to scavenge free radicals [25]. This is particularly true for α-isomers, which show the highest antioxidant activity among tocochromanols.

### 2.2. Tocochromanol Biosynthesis

**Figure 2:**
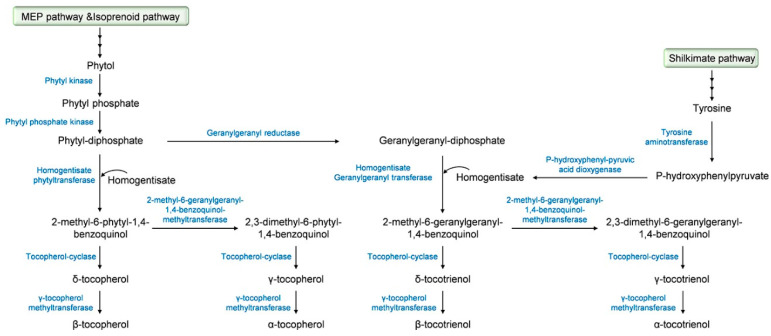
Tocochromanol biosynthetic pathway.

The biosynthetic pathway of tocochromanols has been well documented and we strongly encourage the readers to seek out detailed information in comprehensive studies that have been previously published [24,26,27,28,29,30]. A summary description of this biosynthetic pathway, which starts in the plant cytoplasm for the firsts steps and then takes place in the plastids [31], is provided in Figure 2. The precursor of the tocochromanol biosynthesis is the aromatic head group homogentisate that derives from the catabolism of tyrosine into p-hydroxyphenylpyruvate under the action of p-hydroxyphenyl pyruvic acid dioxygenase. The biosynthesis starts with the condensation of homogentisate with different polyprenyl pyrophosphates that determine the type of tocochromanol, phytyl-diphosphate for tocopherols and geranygeranyl-diphosphate for tocotrienols. Phytyl-diphosphate derives from the methylerytrithol phosphate pathway, more precisely from phytol through the successive action of a phytyl kinase and a phytyl phosphate kinase. Another pathway leading to the production of phytyl-diphosphate is the phytol-recycling pathway derived from the chlorophyll degradation [32,33]. Geranylgeranyl diphosphate is produced from phytyl-diphosphate *via* a reaction catalyzed by the geranylgeranyl reductase. Prenylation of homogentisate with phytyl-diphosphate that leads to the formation of 2-methyl-6-phytyl-1,4-benzoquinol is allowed by the activity of the homogentisate phytyl transferase. For tocotrienols, the prenylation of homogentisate with geranylgeranyl diphosphate is catalyzed by the homogentisate geranylgeranyl transferase that is only located in non-photosynthetic tissues and results in the yield of 2-methyl-6-geranylgeranyl-1,4-benzoquinol [31]. 2-methyl-6-phytyl-1,4-benzoquinol is acted upon by the tocopherol-cyclase to yield δ-tocopherol or by the 2-methyl-6-phytyl-1,4-benzoquinol-methyl transferase, leading to the formation of 2,3-dimethyl-6-phytyl-1,4-benzoquinol and the tocopherol-cyclase to form γ-tocopherol. The final step of tocopherol biosynthesis involves the methylation of δ- and γ-tocopherols to their β- and α- isomers, respectively, under the effect of the γ-tocopherol methyltransferase [34]. α-, β-, δ- and γ-Tocotrienols derive from 2-methyl-6-geranylgeranyl-1,4-benzoquinol, which undergoes the same sequence of methylation and cyclization as 2-methyl-6-phytyl-1,4-benzoquinol with the involvement of the 2-methyl-6-geranylgeranyl-1,4-benzoquinol-methyltransferase, the tocopherol-cyclase, and γ-tocopherol methyltransferase.

Knowledge regarding the key genes involved in tocochromanol biosynthesis has initially focused on *Arabidopsis thaliana*, being facilitated by the access to *Arabidopsis* mutant collections and the possibility to use transgenic plants [35]. These key biosynthetic genes are reported in Table 1. In various cereal crops, including maize, barley, and rice, linkage analysis studies of natural variation for tocochromanol levels have led to the identification of QTLs containing tocochromanol biosynthetic genes in their support intervals [36]. Many of these studies were conducted with a common objective, i.e., improving breeding strategies to increase tocochromanol contents and enriching vegetable oils in vitamin E.

Thus, a genome-wide association approach allowed Schuy et al. [37] to identify two key genes in barley, the homogentisate phytyltransferase (*HPT*) and the homogentisate geranygeranyltransferase (*HGGT*) genes; these two genes were shown to be located on chromosome 7H. In maize, candidate genes encoding the core tocochromanol pathway (*ZmVTE* genes) have also been characterized [28]. Regarding oats, the coding sequences of three genes (*HPPD*, *VTE2* and *VTE4*) have been elucidated [38]. In rice, biosynthetic genes were first identified by comparative genomics with *Arabidopsis thaliana* by Chaudary and Khurana [39], and the function of a few of them (e.g., *OsGGR1* and *OsGGR2*) was further validated using a transgenic approach [40]. The former studies were completed by QTL analyses that have led to clarifying the location of *OsγTMT*—the gene encoding the γ-tocopherol methyltransferase—on chromosome 2 [41].

In addition to genes involved in the biosynthetic pathway, genes or genomic areas associated with the natural variations of tocochromanols have been identified. As an example, the gene *LIL3* coding for a light-harvesting-like protein involved in the stabilization of the geranylgeranyl reductase enzyme is a key actor of the genetic control of tocochromanols in *Arabidopsis thaliana* [42]. Significant insights have also been recently published regarding the genetic determinants that govern the accumulation of tocochromanols in maize; more than 40 genomic areas that do not carry the biosynthetic genes have been related to tocochromanol variations [36]. Among candidate genes identified in the latter study and further supported by the findings of Zhan et al. [43], homologs of protochlorophyllide reductase (*por*) genes (*por1* and *por2*) have been shown to account for large allelic effect for tocochromanol traits. These two genes are known to be involved in the regulation of chlorophyll biosynthesis and their association with tocochromanol variations in maize kernels, i.e., kernels that do not contain chlorophyll, first raised numerous questions. These questions were however rapidly resolved with the demonstration that developing maize embryos contain low levels of chlorophyll [36]. In addition to *por* genes, genes coding for plastid-localized fibrillins and cytosolic glycol lipid transfer and SNARE proteins located on four QTLs (QTL10, QTL30, and QTL6) were also suggested to be responsible for tocochromanol phenotypic variation in maize kernels [36]. In addition, a recent study has indicated the occurrence of genes of the fatty acid pathway in several maize QTLs related to tocochromanol accumulation, suggesting the occurrence of a crosstalk between tocochromanol and fatty acid pathways [44].

### 2.3. Tocochromanol Composition of Major Cereal Crops

**Figure 3:**
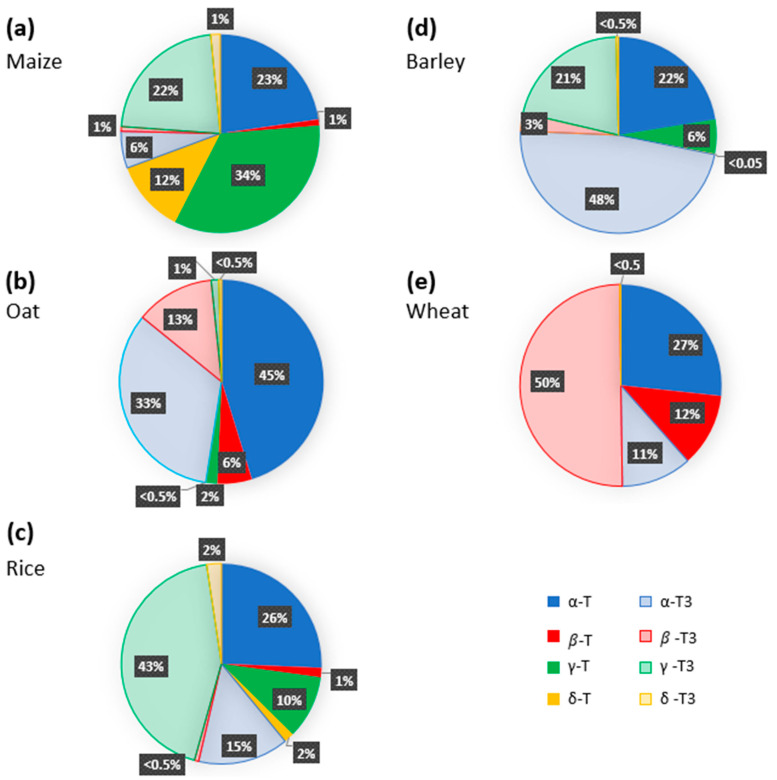
Tocochromanol profile of major cereal crops ((**a**) Maize; (**b**) Oat; (**c**) Rice; (**d**) Barley; (**e**) Wheat). The percentages were calculated using average concentration of tocochromanols reported in previously published studies [38,52,54,56,57,58,59,61,62,63,65,66,67,68].

Numerous studies have addressed the tocochromanol composition in kernels of the main cereal types and analyzed the eight tocochromanol isomers, α-, β-, γ-, and δ-tocopherols and tocotrienols. Data from the most recent publications focusing on oat, wheat, barley, rice and maize kernels are gathered in Figure 3 and Table S1. Before going further in the discussion, it is important to underline that the mean values reported in Figure 3 were calculated using different published reports that considered one or a few genotypes and one or a few years of cultivation in some specific agroclimatic conditions. Considering the significant impact of environmental conditions on tocochromanol levels in cereals [52] and of the genetic background of the cultivar, these data must be taken with caution [28,36,53]. On Figure 3, it appears that all tocochromanol isomers were found in maize, oat, and rice kernels, whereas γ- and δ-tocopherols and γ-tocotrienol in wheat and β-tocopherol in barley were not detected. The highest levels of tocochromanols, 161 µg.g^−1^ dry matter, were reported by Gutierrez-Gonzales et al. in oat kernels [38]. In wheat, rice, maize, and barley kernels, the highest concentration quantified were 69, 60, 41, and 32 µg.g^−1^ dry matter, respectively (Table S1) [52,54,55,56]. When considering the relative distribution between tocotrienols and tocopherols, the tocotrienol/tocopherol ratio (calculated with data presented in Figure 3 and Table S1) was close to 1 for oat indicating equivalent concentrations of tocotrienols and tocopherols and higher than 1 for rice and wheat, reaching 2.5 for barley. In contrast, maize kernels were shown to contain more than 2 times more tocopherols than tocotrienols.

The levels of each tocopherol and tocotrienol isomer quantified in whole kernels vary according to the cereal type (Figure 3). In maize kernels, γ-tocopherol, α-tocopherol, and γ-tocotrienol were the major tocochromanols, which correspond to 34, 23 and 22% of the total tocochromanols (Figure 3a), respectively, and β-tocotrienol, β-tocopherol, δ-tocotrienol, δ-tocopherol, and α-tocotrienol account for 1 to 12% of the total tocochromanol content [56,57,58,59]. A different distribution pattern was observed in small-grain cereals. In oat kernels, α-tocopherol and α-tocotrienol were the prevalent tocochromanols, representing 45% and 33% of the total tocochromanol content, respectively, followed by β-tocotrienol (13%), β-tocopherol (6%), and γ-tocopherol (2%) (Figure 3b) [60,61,62]. The presence of δ-tocotrienol, γ-tocotrienol, and δ-tocopherol was reported as very weak, accounting for less than 1% of the total tocochromanol content. As for oats, the kernels contain the eight tocochromanol isomers, their distribution and concentration being, however, significantly different. In rice, the major tocochromanols are γ-tocotrienol and α-tocopherol, representing, respectively, 43% and 26% of the total tocochromanol content, followed by γ-tocopherol and α-tocotrienol. The sum of δ-tocotrienol, β-tocotrienol, β-tocopherol, and δ-tocopherol accounts for around 5% of the total rice tocochromanol content (Figure 3c) [55,61,63,64,65,66]. In barley kernels, α-tocotrienol is the main compound and accounts for almost half (48%) of the total tocochromanols [52,61,62]. Both α-tocopherol and γ-tocotrienol were estimated to account to 22 and 21% of the total tocochromanol content, respectively (Figure 3d). Regarding wheat kernels, the data reported in Figure 3e indicate that the predominant tocopherol is β-tocotrienol (50%), followed by α-tocopherol (27%) and both β-tocopherol and α-tocotrienol, each of them representing around 11% of the total tocochromanol content [52,54,61,62,67,68].

### 2.4. Distribution of Tocochromanols within Cereal Kernels

Enzymes involved in the biosynthesis of tocopherols are located in the inner membrane of chloroplast envelope and in plastoglobuli [69]. Chloroplasts were first indicated as the unique plant cell organelle containing tocopherols, leading to the mistaken assumption than tocopherols were only present in photosynthetic parts of plants. The occurrence of tocopherols in roots, nuts, and seeds was further evidenced [70,71]. Regarding tocotrienols, they are present in kernels of most monocotyledons, including cereals and absent in the other parts of plants as a result of the specific location of the homogentisate geranygeranyltransferase enzyme in plastid cell of seed endosperms. Data related to the distribution of tochochromanols within cereal kernels are gathered in Table 2. It clearly appears that tocopherols are primarily found in germs, representing more than 89% of the total tocochromanol content; this percentage reaches 98% when considering germs of maize kernels. Moreover, as indicated in Table 2, tocotrienols are predominantly present in endosperm (up to 82% of the total tocochromanol concentration) and in pericarp (up to 78%), except for maize kernels, where the pericarp contains mainly tocopherols. The major isomers found in the different parts of kernels differ depending on the considered cereal. In maize, the γ- isomers of both tocopherol and tocotrienol are predominant in all parts of kernels, with a prevalence of γ-tocopherol in the germ and pericarp (65% and 39%, respectively, of total tocochromanol content) and a majority of γ-tocotrienol in the endosperm (45%) [58,72,73].

In wheat germ, the α- isomer of tocopherol is the most representative tocochromanol, reaching up to 68% of the total, followed by β-tocopherol (25%) and β-tocotrienol (6%). In endosperm and pericarp, β-tocotrienol is the most important tocochromanol (49 and 55%, respectively), followed by γ-tocopherol in endosperm (37%) and α-tocotrienol in pericarp (21%) [54,73,74]. In barley, the α-isomers are the predominant isomers in all parts of the kernels: α-tocopherol in germ (68%) and α-tocotrienol in both endosperm and pericarp (41 and 47%) [73]. In the germ of rice kernels, α-tocopherol is largely predominant, accounting for more than 80% of the total tocochromanol content. In the endosperm of rice kernels, α-tocopherol and γ-tocotrienol are equally represented (39%) followed by α-tocotrienol (21%), while in pericarp α-tocopherol is the most represented tocochromanol (37%) followed by γ-tocotrienol and α-tocotrienol (29 and 27%, respectively) [73].

### 2.5. Kinetics of Tocochromanol Accumulation during Maturation of Cereal Kernels

There are very few dynamic studies that have addressed the tocochromanol composition of kernels from the early stages of kernel development until maturity. This knowledge is, however, essential to clarify which compounds fungal pathogens face at the onset of infection and to support their potential role in plant defense. In maize, several studies corroborate an increase of tocochromanol content starting from pollination. In the report of Xie et al. [56], focusing on the first 30 days following pollination of maize, a regular increase of both tocopherol and tocotrienol was described. Increase in tocopherol content was shown to continue until 96 days after silking by Picot et al. [75]. In oat kernels, a pronounced increase in tocochromanols was reported from 14 days after anthesis to the maturity stage [60]. In barley, tocochromanols were reported to be at their highest concentration at a very early stage (milk stage) to then slightly decrease until maturity [76]. In rice, the kinetics of tocochromanol content during seed filling were significantly different between tocopherols and tocotrienols; tocopherols were reported to decrease, while an inverse tendency was described for tocotrienols [66].

## 3. Tocochromanols as Part of the Plant Chemical Defense Arsenal against Phytopathogenic and Toxigenic Fungi

Whereas the contribution of tocochromanols to plant protection against various abiotic stresses has been widely studied, relatively little is known regarding their potential implication in plant defense against pathogens. Moreover, the available knowledge on physiological roles of tocochromanols in plants is mainly related to tocopherols; studies on tocotrienols are much more recent, with first hypotheses on their functional role released in 2008 [77]. Comparison of tocopherol contents in control plants *versus* plants submitted to environmental stresses or use of mutants altered in their tocopherol content, has led to suggest a significant role of tocopherols in plant tolerance to drought, cold, high light, heavy metal, and salinity stress [78,79,80,81]. For a comprehensive and in-depth view of the various functions tocochromanols could play in plants, we strongly encourage readers to consult the reviews of Munné-Bosch [69] and Méné-Saffrané and DellaPenna [26]. One of the key mechanisms underlying the capacity of tocochromanols to mitigate the plant damage induced by abiotic stresses is related to their ability to scavenge or quench lipid peroxides, oxygen radicals, or singlet oxygen [82]. In addition to their antioxidant function, tocochromanols have also been suggested to interfere with cellular signaling by interacting with plant hormones (ethylene, abscisic acid, salicylic acid, and jasmonic acid) and sugar regulatory networks [80,83,84]. More recent studies have also reported a possible role of tocopherols as regulators of miRNA biogenesis, miRNA being key players in posttranscriptional regulation of gene expression during plant adaptation to stresses [85].

**Figure 4:**
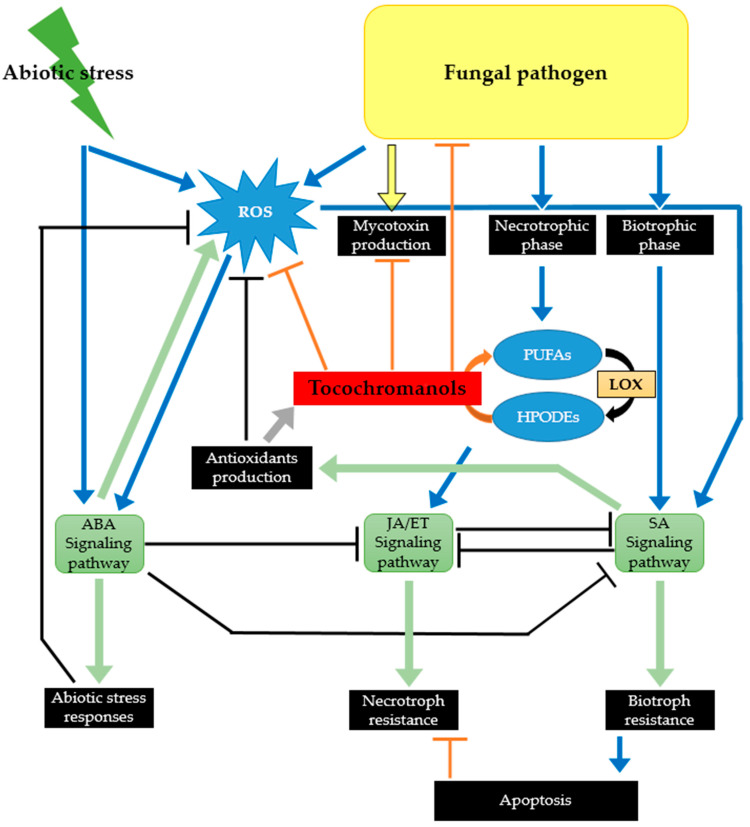
Involvement of tocochromanols in plant response to biotic and abiotic stresses. ROS: reactive oxygen species; PUFAs: polyunsaturated fatty acids; HPODEs: hydroperoxides; LOX: lipoxygenase; ABA: abscisic acid; JA: jasmonic acid; ET: ethylene; SA: salicylic acid.

Though understudied, the importance of tocochromanols in plant resistance to pathogens is strongly supported by the accumulated knowledge on their role in plant protection to abiotic stresses. Indeed, biotic and abiotic stress responses cannot be dissociated, their interactions being predominantly orchestrated by plant hormones [86]. Moreover, similarly to abiotic stresses effects, biotic ones lead to an increased accumulation of ROS in plant tissues. In previously published studies addressing the involvement of plant metabolites in the mechanisms employed by crops to counteract fungal pathogens, tocochromanols are almost never mentioned [4,87]. One rationale for this could be related to the insufficient coverage of metabolomics approaches applied to decipher the chemical defense of plants that frequently excludes lipophilic compounds [4]. Nonetheless, we are convinced that tocochromanols, as part of plant protection to biotic stresses, are not receiving the attention they deserve. In the following section, the reasons why this class of compound should not be overlooked when dealing with crop resistance to fungal pathogens and more specifically to toxigenic ones is discussed. The discussed body of arguments is schematized in Figure 4.

### 3.1. By Protecting Crops against Environmental Stresses, Tocochromanols Can Indirectly Affect Their Susceptibility to Fungal Pathogens

Environmental factors are acknowledged to modulate the interactions between plant and fungal pathogens leading to either an increase or a decrease in plant susceptibility to fungal disease, depending on the nature, timing and severity of both the environmental constraint and the pathogen [88]. The widely accepted belief that climate change will have a severe impact on the development of toxigenic fungi in crops and consequently on the contamination of harvests with mycotoxins has led to increased research efforts on the effect of drought and temperature elevation on fungal population migration and fungal species adaptation [89], but the way climate change will affect plant–toxigenic fungi interactions remains today largely insufficiently investigated. Hence, previous reports have suggested that wheat plants exposed to a drought stress were more susceptible to deoxynivalenol-producing *Fusarium* strains [90,91]. Similarly, contamination with aflatoxin and fumonisin mycotoxins have been reported to be worsened in maize plants cultivated under drought conditions [92,93]. Accordingly, the results of Abbas et al. [94] indicated that maize genotypes less susceptible to high temperature and drought were less prone to contamination with mycotoxins. The previous observations focusing on toxigenic fungi are supported by recent reviews published by Desaint et al. [95] and Pandey et al. [88] indicating that in half of the reported cases, temperature elevation and drought led to an increased plant susceptibility to the pathogen and/or a to a decrease efficacy of plant defense strategies. The molecular mechanisms underlying the negative effect of elevated temperature and drought stress on plant resistance to toxigenic fungi are not yet fully elucidated. Among probable assumptions, one can mention: (i) the role of reactive oxygen species accumulating in plant tissues in response to abiotic stresses and known to stimulate the yield of mycotoxins by toxigenic fungi [96], (ii) the role of stress-related amino acids such as asparagine or putrescine that have also been reported to promote the production of some mycotoxins [97,98], and (iii) the role of phytohormones and derived signaling pathways reported as key mediators of combined stress responses [99]. Consequently, by their capacity to mitigate oxidative stress in plant tissues and also to interfere with phytohormone regulatory networks, it is highly likely that tocochromanols can mitigate the negative impact environmental stresses can have on crop susceptibility to diseases caused by toxigenic fungi.

### 3.2. Tocochromanols Can Mitigate the Damage Caused by ROS Produced in Response to Infection by Toxigenic Fungal Pathogens

The key role played by ROS in plant–fungal pathogen interactions has been extensively reviewed [15,100,101]. A rapid, transient, production of huge amounts of ROS (including singlet oxygen, superoxide ions, hydrogen peroxide, and hydroxyl radicals) is one of the earliest mechanisms of plant defense strategies against pathogens. Such an oxidative burst has been observed in wheat inoculated with a deoxynivalenol-producing *Fusarium graminearum* isolate [102,103] and, more recently, a *Fusarium culmorum* strain [104]. The evidence of increased expression of antioxidant mechanisms in maize inoculated with *Aspergillus flavus* [105] or *Fusarium verticillioides* [106] also significantly supports the occurrence of an oxidative burst following infection with these two major toxigenic fungi affecting maize crops. ROS accumulation acts as a double-edged sword in plant defense cellular processes. On one hand, ROS function as signal transducers activating local as well as systemic plant defense machinery. On the other hand, when the timing and magnitude of ROS accumulation are not tightly controlled by the plant’s antioxidant system, including enzymatic and non-enzymatic components, ROS can lead to cellular damage such as the degradation of membrane lipids, proteins, and DNA and ultimately to cellular death. Such necrosis of plant tissues can increase host susceptibility to necrotrophic fungi that feed from dead plant material and include most of the toxigenic fungi–infecting crops. Indeed, the main aflatoxin producing species, i.e., *A. flavus* and *Aspergillus parasiticus*, are recognized as necrotrophic fungal agents [107]. In addition, the infection strategy of *Fusarium* species capable of producing mycotoxins, including *F. graminearum*, *F. culmorum*, *F. verticillioides,* and *Fusarium proliferatum*, is also characterized by the occurrence of a necrotrophic phase. Actually, toxigenic *Fusarium* species are hemi-biotrophic fungi: they first infect living plant tissues as biotrophs but after a short latency period switch to necrotrophic growth [108]. Moreover, there is converging evidence indicating that the redox balance is a key factor regulating the mycotoxin biosynthesis and that exposure to ROS triggers and/or increases the production of mycotoxins by fungi (reviewed in Montibus et al. [109] and Gil-Serna et al. [110]). All together, the aforementioned data indicate that ROS-overproduction, triggered by the infection by toxigenic fungi as an early plant defense mechanism, could in fact be used by the fungal pathogen to its own benefit. To cope with oxidative stress, plants have evolved diverse enzymatic and non-enzymatic machineries. In addition to specialized metabolites such as carotenoids and flavonoids, tocochromanols, which act cooperatively with ascorbate and glutathione, are acknowledged as key components of the plant antioxidant non-enzymatic machinery [111]. The capacity of tocochromanols to scavenge excess ROS and, in particular, singlet oxygen has been extensively studied [112]. For a detailed view on the structural determinants but also on the physical and chemical mechanisms involved in singlet oxygen quenching by tocochromanols, we strongly encourage the reader to read the review of Kamal-Eldin and Appelqvist [17]. Therefore, by contributing to alleviate ROS levels surrounding invasive hyphae during plant infection, tocochromanols are likely to mitigate the positive control exerted by oxidative stress on toxigenic fungal development and mycotoxin production.

### 3.3. Tocochromanols Can Interfere with Fatty Acid Metabolism and Consequently with Plant Signaling Networks

The major phytohormones are abscisic acid, auxin, brassinosteroids, cytokinins, gibberellins, ethylene, jasmonic acid, salicylic acid, and strigolactones. Among these, jasmonic acid, salicylic acid, and ethylene are known to play central roles in orchestrating plant defense responses against various pathogens [113,114]. Even though these three phytohormones intimately interact, the salicylic acid signaling pathway is mainly associated with activation of defense response against biotrophic and hemi-biotrophic fungal pathogens, whereas the jasmonic acid and ethylene signaling pathways are mostly involved in defense against necrotrophic fungi [113]. Salicylic and jasmonic acids have been reported to significantly and positively contribute to wheat resistance to the hemi-biotrophic *F. graminearum* pathogen, while ethylene was associated with wheat susceptibility [115]. According to Makandar et al. [116], the role of the jasmonate signaling pathway in the interaction of wheat with *F. graminearum* is more contrasted: jasmonic acid could promote disease by constraining the salicylic acid signaling pathway during the early biotrophic stage of infection and promote resistance during the later necrotrophic stage. Involvement of jasmonic acid and methyl jasmonate in resistance to deoxynivalenol-producing *Fusarium* isolates has been evidenced by the comparison of *Fusarium*-inoculated and mock-inoculated wheat ear metabolomic profiles [4] and corroborated by transcriptional studies [115,117]. Moreover, according to Gunnaiah et al. [118], deoxynivalenol application on wheat tissue also led to an induction of jasmonate signaling. The key contribution of jasmonic and salicylic acid to plant resistance to toxigenic fungi has also been suggested for maize and various toxigenic fungal pathogens, including the fumonisin producer *F. verticillioides* [119] and the aflatoxin producing *A. flavus* species [120]. The expression of a wide set of defense genes has been shown to be regulated by jasmonic acid and methyl jasmonate, including genes related to specialized metabolites pathways such as phenylpropanoids or terpenoids, genes coding for defensive proteins (pathogenesis-related or PR proteins), and genes related to redox homeostasis [121]. Jasmonic acid signaling has also been reported to influence callose deposition, which in wheat is a key mechanism dampening *F. graminearum* spread within the ear [122]. Moreover, external application of jasmonic acid has been shown to activate glucosyltransferase in barley [123], which is a key enzyme activity involved in a deoxynivalenol detoxification pathway that transforms the toxin into the less phytotoxic deoxynivalenol-3-O-glucoside. Jasmonic acid belongs to the family of oxylipins that gathers lipophilic signaling molecules derived from the oxidation of polyunsaturated fatty acids. In fact, jasmonic acid is synthesized from α-linolenic acid (18:3) following a process of oxidation, cyclisation and acyl chain shortening [121]. The first step of its biosynthetic pathway is the conversion of α-linolenic acid to 13-hydroperoxylinolenic acid by a 13-lipoxygenase. Considering the widely acknowledged capacity of tocochromanols to scavenge lipid peroxyl radicals and lower the extent of lipid peroxidation [84,124] but also their potential to affect lipoxygenase activity through competitive inhibition [125,126], it is more than likely that tocochromanols can negatively affect the biosynthesis of jasmonic acid and, in consequence, modulate the jasmonic acid-mediated response of plants. Similarly, tocochromanols are theoretically able to reduce the amounts of other lipid oxidation products such as those derived from the action of 9-lipoxygenase that, even though less studied, could also be key players of plant response to fungal attacks. In several studies, products generated by 9-lipoxygenase were suggested to contribute to the susceptibility of the plant host. Thus, Gao et al. [127] observed an increased susceptibility of maize to *A. flavus* and *Aspergillus nidulans* but also to *F. verticillioides* in maize mutant lines in which the function of a 9-lipoxygenase gene was abolished. A similar conclusion was raised by Nalam et al. [128] who observed that the silencing of a gene coding for a 9-lipoxygenase in wheat led to an enhanced susceptibility to *F. graminearum*. The oxylipin-mediated crosstalk between plant host and toxigenic fungi has been the subject of extensive research during the two past decades. There has been growing evidence that oxylipins can modulate spore production, fungal development, reproduction and secondary metabolites biosynthesis [127,129,130]. Investigations focusing on the biosynthesis of aflatoxins and fumonisins have demonstrated the opposite effect of 13-lipoxygenase and 9-lipoxygenase oxidation products (recently reviewed in Qiu et al. [131]). Whereas fatty acid hydroperoxides generated through the 9-lipoxygenase pathway were shown to mimic the fungal oxylipins called psi factors for precocious sexual inducers and promote the production of mycotoxins, products derived from the action of 13-lipoxygenase exhibited a significant inhibitor effect [132,133]. Less is known regarding the effect of oxylipins on the production of deoxynivalenol by *F. graminearum* and *F. culmorum*. The hypothesis that 9-lipoxygenase hydroperoxides could be toxin-conducive factors in wheat raised by Nobili et al. [133] and Nalam et al. [128] requires still to be demonstrated. Lastly, as highlighted for *F. verticillioides* [134], oxylipins derived from additional lipoxygenase pathways such as those related to the 3, 4 and 5-lipoxygenase in maize are also strongly suspected to interfere with fungal colonization and its production of mycotoxins.

### 3.4. Tocochromanols Display Antifungal and Antimycotoxin Activities

As lipophilic components, tocochromanols are thought to induce perturbations in the membrane of microorganisms, to possibly disrupt its integrity and affect the cell survival. This bioactivity has been mainly studied against bacteria pathogenic to humans. The capacity of α-tocopherol to inhibit the growth of *Staphylococcus aureus*, *Pseudomonas aeruginosa* and *Escherichia coli* was reported by Andrade et al. [135] and supported by the characterization of the antimicrobial activity of *Codonopsis lanceolate* plants enriched in α-tocopherol through overexpression of the γ-tocopherol methyltransferase [136]. Regarding fungal pathogens, very few studies have investigated the effect of tocochromanols on the growth of toxigenic fungi and mycotoxin production. Most of the arguments that substantiate the hypothesis of an antifungal or/and an antimycotoxin effect comes from studies investigating the relationships between the bioactivity of plant extracts and their richness in tocochromanols [137,138]. To our knowledge, the only studies addressing the direct effect of tocochromanols on mycotoxin yield are the reports of Norton [139] and Picot et al. [75]. The results of the two previous studies showed, however, high discrepancies; while aflatoxin and *A. flavus* growth were not affected by supplementation with α- and γ-tocopherols, at concentrations ranging from 0.05 to 5 mM [139,140], a significant inhibition of the production of fumonisins by *F. verticillioides* was induced by 0.01 mM of α-tocopherol [75]. These inconsistencies clearly show the need to deepen the investigation of tocochromanol bioactivity against toxigenic fungi.

## 4. Conclusions

To cope with fungal pathogens, cereals have developed a variety of biochemical responses to avoid infection and reduce adverse effects, including the contamination of ears with mycotoxins. Whereas numerous reports have appeared in the scientific literature over the past several years regarding the role of phenylpropanoids and terpenoids in plant chemical defense, the potential contribution of tocochromanols has been scarcely addressed. Yet, as emphasized by the information compiled in the present review, tocochromanols are widely distributed in cereals, and there are strong arguments in favor of a pivotal role played by these compounds. These arguments include: (i) their capacity to alleviate ROS-induced oxidative damage, (ii) their capacity to interfere with plant hormone signaling pathways, and (iii) their potential to reduce fungal growth and mycotoxin yield. An exciting challenge for the near future is therefore to validate this hypothesis as this could open the way to help breeders to select cereal genotypes less susceptible to contamination with mycotoxins and ensure the safety of harvests. The currently available knowledge regarding the biosynthetic pathway of tocochromanols and associated genes will be a significant asset to foster scientific research on the way tocochromanols assist cereals to combat toxigenic fungi.

## Figures and Tables

**Table 1:** Key genes involved in tocochromanol biosynthesis in *Arabidopsis thaliana* and various cereal crops.

Plant Species	Gene	Enzyme ^1^	Ref.
*Arabidopsis thaliana*			
	*PSD1*	HPPD	[45]
	*GGR*	GGR	[46]
	*VTE1*	Tocopherol cyclase	[35]
	*VTE2*	HPT	[35]
	*VTE3*	MPBQ/MGGBQ MT	[47]
	*VTE4*	γ-TMT	[34]
	*VTE5*	Phytol kinase	[32]
	*VTE6*	Phytyl-P kinase	[33]
	*HGGT*	HGGT	[48]
Barley			
	*VTE1*	Tocopherol cyclase	[49]
	*VTE4*	γ-TMT	[49]
	*HPT-7H*	HPT	[37]
	*HGGT*	HGGT	[37]
Maize			
	*ZmVTE1*	Tocopherol cyclase	[28]
	*ZmVTE2*	HPT	[28]
	*ZmVTE3*	MPBQ/MGGBQ MT	[28]
	*ZmVTE4*	γ-TMT	[28]
	*ZmVTE5*	Phytol kinase	[28]
	*ZmHPPD*	HPPD	[28]
Rice			
	*OsGGR1*	GGR	[40]
	*OsGGR2*	GGR	[40]
	*OsγTMT*	γ-TMT	[41]
	*SGD1*	HPT	[50]
	*RTD1*	HPT	[51]
Oat			
	*HPPD (3 homo)*	HPPD	[38]
	*VTE2_3/VTE2_4*	HPT	[38]
	*VTE4_1*	γ-TMT	[38]

^1^ HPPD: p-hydroxyphenyl pyruvic acid dioxygenase; GGR: geranylgeranyl reductase; HPT: homogentisate phytyltransferase; MPBQ: 2-methyl-6-phytyl-1,4-benzoquinol; MGGBQ: 2-methyl-6-geranylgeranyl-1,4-benzoquinol; MT: methyltransferase; γ-TMT: γ-tocopherol methyltransferase; Phytyl-P kinase: phytyl phosphate kinase; HGGT: homogentisate geranylgeranyltransferase.

**Table 2:** Repartition of tocochromanols in the different parts of cereal kernels.

	α-T ^1^ (%)	β-T (%)	γ-T (%)	δ-T (%)	α-T3 ^1^ (%)	β-T3 (%)	γ-T3 (%)	δ-T3 (%)	Total Tocopherol (%)	Total Tocotrienol (%)
Maize										
Germ	30	1	65	3	1	0	1	0	98	2
Endosperm	5	0	19	0	29	0	45	2	24	76
Pericarp	18	0	52	6	9	1	14	1	75	25
Wheat										
Germ	69	26	0	0	2	3	ND ^2^	0	95	5
Endosperm	5	3	13	0	11	68	ND	0	21	79
Pericarp	7	3	8	0	21	61	ND	0	18	82
Barley										
Germ	68	3	16	1	6	2	3	0	89	11
Endosperm	14	1	2	1	41	25	15	3	17	83
Pericarp	15	1	5	1	47	12	17	3	21	79
Rice										
Germ	81	3	5	0	6	ND	4	0	89	11
Endosperm	33	2	6	2	21	ND	33	4	42	58
Pericarp	37	1	4	0	27	ND	29	2	43	57

^1^ T corresponds to tocopherols and T3 corresponds to tocotrienols; ^2^ ND = not detected. The percentages reported in Table 2 correspond to the average values of distribution percentages reported in previously published reports [54,58,72,73,74].

## Data Availability

Not applicable.

## Supplementary Materials

The following supporting information can be downloaded at: https://www.mdpi.com/article/10.3390/ijms23169303/s1.

## References

[1] OECD Agriculture Statistics. https://www.oecd-ilibrary.org/agriculture-and-food/data/oecd-agriculture-statistics_agr-data-en.

[2] Savary S., Willocquet L., Pethybridge S.J., Esker P., McRoberts N., Nelson A. (2019). The Global Burden of Pathogens and Pests on Major Food Crops. Nat. Ecol. Evol..

[3] Zaynab M., Mahpara F., Safdar A., Yasir S., Muhammad U., Muhammad H.Z., Khalida B. (2018). Role of Secondary Metabolites in Plant Defense against Pathogens. Microb. Pathog..

[4] Gauthier L., Atanasova-Penichon V., Chéreau S., Richard-Forget F. (2015). Metabolomics to Decipher the Chemical Defense of Cereals against Fusarium Graminearum and Deoxynivalenol Accumulation. Int. J. Mol. Sci..

[5] Agati G., Azzarello E., Pollastri S., Tattini M. (2012). Flavonoids as Antioxidants in Plants: Location and Functional Significance. Plant Sci..

[6] Vincenzo L., Veronica M.T.L., Angela C., Imperato F. (2006). Role of Phenolics in the Resistance Mechanisms of Plants against Fungal Pathogens and Insects. Phytochemistry: Advances in Research.

[7] Sattler S.E., Funnell-Harris D.L. (2013). Modifying Lignin to Improve Bioenergy Feedstocks: Strengthening the Barrier against Pathogens?. Front. Plant Sci..

[8] Atanasova-Penichon V., Barreau C., Richard-Forget F. (2016). Antioxidant Secondary Metabolites in Cereals: Potential Involvement in Resistance to Fusarium and Mycotoxin Accumulation. Front. Microbiol..

[9] Bollina V., Kushalappa A.C., Choo T.M., Dion Y., Rioux S. (2011). Identification of Metabolites Related to Mechanisms of Resistance in Barley against Fusarium Graminearum, Based on Mass Spectrometry. Plant Mol. Biol..

[10] Boncan D.A.T., Tsang S.S.K., Li C., Lee I.H.T., Lam H.-M., Chan T.-F., Hui J.H.L. (2020). Terpenes and Terpenoids in Plants: Interactions with Environment and Insects. Int. J. Mol. Sci..

[11] Abbas F., Ke Y., Yu R., Yue Y., Amanullah S., Jahangir M.M., Fan Y. (2017). Volatile Terpenoids: Multiple Functions, Biosynthesis, Modulation and Manipulation by Genetic Engineering. Planta.

[12] Schmelz E.A., Huffaker A., Sims J.W., Christensen S.A., Lu X., Okada K., Peters R.J. (2014). Biosynthesis, Elicitation and Roles of Monocot Terpenoid Phytoalexins. Plant J. Cell Mol. Biol..

[13] Mauch-Mani B., Mauch F. (2005). The Role of Abscisic Acid in Plant–Pathogen Interactions. Curr. Opin. Plant Biol..

[14] Robert-Seilaniantz A., Navarro L., Bari R., Jones J.D. (2007). Pathological Hormone Imbalances. Curr. Opin. Plant Biol..

[15] Dumanović J., Nepovimova E., Natić M., Kuča K., Jaćević V. (2021). The Significance of Reactive Oxygen Species and Antioxidant Defense System in Plants: A Concise Overview. Front. Plant Sci..

[16] Santos L., Kasper R., Gil-Serna J., Marín S., Sanchis V., Ramos A.J. (2010). Effect of Capsicum Carotenoids on Growth and Ochratoxin A Production by Chilli and Paprika Aspergillus Spp. Isolates. Int. J. Food Microbiol..

[17] Kamal-Eldin A., Appelqvist L.-Å. (1996). The Chemistry and Antioxidant Properties of Tocopherols and Tocotrienols. Lipids.

[18] Hernández I., Alegre L., Munné-Bosch S. (2004). Drought-Induced Changes in Flavonoids and Other Low Molecular Weight Antioxidants in Cistus Clusii Grown under Mediterranean Field Conditions. Tree Physiol..

[19] Chennupati P., Seguin P., Liu W. (2011). Effects of High Temperature Stress at Different Development Stages on Soybean Isoflavone and Tocopherol Concentrations. J. Agric. Food Chem..

[20] Kodad O., Socias i Company R., Alonso J.M. (2018). Genotypic and Environmental Effects on Tocopherol Content in Almond. Antioxidants.

[21] Xiang N., Li C., Li G., Yu Y., Hu J., Guo X. (2019). Comparative Evaluation on Vitamin E and Carotenoid Accumulation in Sweet Corn (*Zea mays* L.) Seedlings under Temperature Stress. J. Agric. Food Chem..

[22] Asensi-Fabado M.A., Ammon A., Sonnewald U., Munné-Bosch S., Voll L.M. (2015). Tocopherol Deficiency Reduces Sucrose Export from Salt- Stressed Potato Leaves Independently of Oxidative Stress and Symplastic Obstruction by Callose. J. Exp. Bot..

[23] Kruk J., Holländer-Czytko H., Oettmeier W., Trebst A. (2005). Tocopherol as Singlet Oxygen Scavenger in Photosystem II. J. Plant Physiol..

[24] Muñoz P., Munné-Bosch S. (2019). Vitamin E in Plants: Biosynthesis, Transport, and Function. Trends Plant Sci..

[25] Müller L., Fröhlich K., Böhm V. (2011). Comparative Antioxidant Activities of Carotenoids Measured by Ferric Reducing Antioxidant Power (FRAP), ABTS Bleaching Assay (ATEAC), DPPH Assay and Peroxyl Radical Scavenging Assay. Food Chem..

[26] Mène-Saffrané L., DellaPenna D. (2010). Biosynthesis, Regulation and Functions of Tocochromanols in Plants. Plant Physiol. Biochem..

[27] Hussain N., Irshad F., Jabeen Z., Shamsi I.H., Li Z., Jiang L. (2013). Biosynthesis, Structural, and Functional Attributes of Tocopherols in Planta; Past, Present, and Future Perspectives. J. Agric. Food Chem..

[28] Lipka A.E., Gore M.A., Magallanes-Lundback M., Mesberg A., Lin H., Tiede T., Chen C., Buell C.R., Buckler E.S., Rocheford T. (2013). Genome-Wide Association Study and Pathway-Level Analysis of Tocochromanol Levels in Maize Grain. G3 GenesGenomesGenetics.

[29] Fritsche S., Wang X., Jung C. (2017). Recent Advances in Our Understanding of Tocopherol Biosynthesis in Plants: An Overview of Key Genes, Functions, and Breeding of Vitamin E Improved Crops. Antioxidants.

[30] Ma J., Qiu D., Pang Y., Gao H., Wang X., Qin Y. (2020). Diverse Roles of Tocopherols in Response to Abiotic and Biotic Stresses and Strategies for Genetic Biofortification in Plants. Mol. Breed..

[31] Mène-Saffrané L. (2017). Vitamin E Biosynthesis and Its Regulation in Plants. Antioxidants.

[32] Valentin H.E., Lincoln K., Moshiri F., Jensen P.K., Qi Q., Venkatesh T.V., Karunanandaa B., Baszis S.R., Norris S.R., Savidge B. (2006). The Arabidopsis Vitamin E Pathway Gene5-1 Mutant Reveals a Critical Role for Phytol Kinase in Seed Tocopherol Biosynthesis. Plant Cell.

[33] Vom Dorp K., Hölzl G., Plohmann C., Eisenhut M., Abraham M., Weber A.P., Hanson A.D., Dörmann P. (2015). Remobilization of Phytol from Chlorophyll Degradation Is Essential for Tocopherol Synthesis and Growth of Arabidopsis. Plant Cell.

[34] Bergmüller E., Porfirova S., Dörmann P. (2003). Characterization of an Arabidopsis Mutant Deficient in γ-Tocopherol Methyltransferase. Plant Mol. Biol..

[35] Porfirova S., Bergmüller E., Tropf S., Lemke R., Dörmann P. (2002). Isolation of an Arabidopsis Mutant Lacking Vitamin E and Identification of a Cyclase Essential for All Tocopherol Biosynthesis. Proc. Natl. Acad. Sci. USA.

[36] Diepenbrock C.H., Kandianis C.B., Lipka A.E., Magallanes-Lundback M., Vaillancourt B., Góngora-Castillo E., Wallace J.G., Cepela J., Mesberg A., Bradbury P.J. (2017). Novel Loci Underlie Natural Variation in Vitamin E Levels in Maize Grain. Plant Cell.

[37] Schuy C., Groth J., Ammon A., Eydam J., Baier S., Schweizer G., Hanemann A., Herz M., Voll L.M., Sonnewald U. (2019). Deciphering the Genetic Basis for Vitamin E Accumulation in Leaves and Grains of Different Barley Accessions. Sci. Rep..

[38] Gutierrez-Gonzalez J.J., Garvin D.F. (2016). Subgenome-Specific Assembly of Vitamin E Biosynthesis Genes and Expression Patterns during Seed Development Provide Insight into the Evolution of Oat Genome. Plant Biotechnol. J..

[39] Chaudhary N., Khurana P. (2009). Vitamin E Biosynthesis Genes in Rice: Molecular Characterization, Expression Profiling and Comparative Phylogenetic Analysis. Plant Sci..

[40] Kimura E., Abe T., Murata K., Kimura T., Otoki Y., Yoshida T., Miyazawa T., Nakagawa K. (2018). Identification of OsGGR2, a Second Geranylgeranyl Reductase Involved in α-Tocopherol Synthesis in Rice. Sci. Rep..

[41] Wang X.-Q., Yoon M.-Y., He Q., Kim T.-S., Tong W., Choi B.-W., Lee Y.-S., Park Y.-J. (2015). Natural Variations in OsγTMT Contribute to Diversity of the α-Tocopherol Content in Rice. Mol. Genet. Genom..

[42] Tanaka R., Rothbart M., Oka S., Takabayashi A., Takahashi K., Shibata M., Myouga F., Motohashi R., Shinozaki K., Grimm B. (2010). LIL3, a Light-Harvesting-like Protein, Plays an Essential Role in Chlorophyll and Tocopherol Biosynthesis. Proc. Natl. Acad. Sci. USA.

[43] Zhan W., Liu J., Pan Q., Wang H., Yan S., Li K., Deng M., Li W., Liu N., Kong Q. (2019). An Allele of ZmPORB2 Encoding a Protochlorophyllide Oxidoreductase Promotes Tocopherol Accumulation in Both Leaves and Kernels of Maize. Plant J..

[44] Wang H., Xu S., Fan Y., Liu N., Zhan W., Liu H., Xiao Y., Li K., Pan Q., Li W. (2018). Beyond Pathways: Genetic Dissection of Tocopherol Content in Maize Kernels by Combining Linkage and Association Analyses. Plant Biotechnol. J..

[45] Norris S.R., Shen X., Della Penna D. (1998). Complementation of the Arabidopsis Pds1 Mutation with the Gene Encoding P-Hydroxyphenylpyruvate Dioxygenase. Plant Physiol..

[46] Keller Y., Bouvier F., d’Harlingue A., Camara B. (1998). Metabolic Compartmentation of Plastid Prenyllipid Biosynthesis. Eur. J. Biochem..

[47] Cheng Z., Sattler S., Maeda H., Sakuragi Y., Bryant D.A., DellaPenna D. (2003). Highly Divergent Methyltransferases Catalyze a Conserved Reaction in Tocopherol and Plastoquinone Synthesis in Cyanobacteria and Photosynthetic Eukaryotes. Plant Cell.

[48] Zhang C., Cahoon R.E., Hunter S.C., Chen M., Han J., Cahoon E.B. (2013). Genetic and Biochemical Basis for Alternative Routes of Tocotrienol Biosynthesis for Enhanced Vitamin E Antioxidant Production. Plant J..

[49] Graebner R.C., Wise M., Cuesta-Marcos A., Geniza M., Blake T., Blake V.C., Butler J., Chao S., Hole D.J., Horsley R. (2015). Quantitative Trait Loci Associated with the Tocochromanol (Vitamin E) Pathway in Barley. PLoS ONE.

[50] Wang D., Wang Y., Long W., Niu M., Zhao Z., Teng X., Zhu X., Zhu J., Hao Y., Wang Y. (2017). SGD1, a Key Enzyme in Tocopherol Biosynthesis, Is Essential for Plant Development and Cold Tolerance in Rice. Plant Sci. Int. J. Exp. Plant Biol..

[51] Yunhui Z., Kai L., Xiaomei Z., Yan W., Suobing Z., Haiyuan C., Jing L., Yingjie W., Xianwen F. (2018). Rice Tocopherol Deficiency 1 Encodes a Homogentisate Phytyltransferase Essential for Tocopherol Biosynthesis and Plant Development in Rice. Plant Cell Rep..

[52] Lachman J., Hejtmánková A., Orsák M., Popov M., Martinek P. (2018). Tocotrienols and Tocopherols in Colored-Grain Wheat, Tritordeum and Barley. Food Chem..

[53] Sookwong P., Murata K., Nakagawa K., Shibata A., Kimura T., Yamaguchi M., Kojima Y., Miyazawa T. (2009). Cross-Fertilization for Enhancing Tocotrienol Biosynthesis in Rice Plants and QTL Analysis of Their F2 Progenies. J. Agric. Food Chem..

[54] Hidalgo A., Brandolini A. (2008). Protein, Ash, Lutein and Tocols Distribution in Einkorn (*Triticum monococcum* L. Subsp. Monococcum) Seed Fractions. Food Chem..

[55] Goufo P., Trindade H. (2014). Rice Antioxidants: Phenolic Acids, Flavonoids, Anthocyanins, Proanthocyanidins, Tocopherols, Tocotrienols, γ-Oryzanol, and Phytic Acid. Food Sci. Nutr..

[56] Xie L., Yu Y., Mao J., Liu H., Hu J., Li T., Guo X., Liu R. (2017). Evaluation of Biosynthesis, Accumulation and Antioxidant Activity of Vitamin E in Sweet Corn (*Zea mays* L.) during Kernel Development. Int. J. Mol. Sci..

[57] Kurilich A.C., Juvik J.A. (1999). Quantification of Carotenoid and Tocopherol Antioxidants in *Zea mays*. J. Agric. Food Chem..

[58] Sun X., Ma L., Lux P.E., Wang X., Stuetz W., Frank J., Liang J. (2022). The Distribution of Phosphorus, Carotenoids and Tocochromanols in Grains of Four Chinese Maize (*Zea mays* L.) Varieties. Food Chem..

[59] Franzen J., Haaß M.M. (1991). Vitamin E Content during Development of Some Seedlings. Phytochemistry.

[60] Gutierrez-Gonzalez J.J., Wise M.L., Garvin D.F. (2013). A Developmental Profile of Tocol Accumulation in Oat Seeds. J. Cereal Sci..

[61] Horvath G., Wessjohann L., Bigirimana J., Jansen M., Guisez Y., Caubergs R., Horemans N. (2006). Differential Distribution of Tocopherols and Tocotrienols in Photosynthetic and Non-Photosynthetic Tissues. Phytochemistry.

[62] Zieliński H., Ciska E., Kozlowska H. (2001). The Cereal Grains: Focus on Vitamin E. Czech J. Food Sci..

[63] Yu L., Li G., Li M., Xu F., Beta T., Bao J. (2016). Genotypic Variation in Phenolic Acids, Vitamin E and Fatty Acids in Whole Grain Rice. Food Chem..

[64] Bergman C.J., Xu Z. (2003). Genotype and Environment Effects on Tocopherol, Tocotrienol, and γ-Oryzanol Contents of Southern U.S. Rice. Cereal Chem..

[65] Heinemann R.J.B., Xu Z., Godber J.S., Lanfer-Marquez U.M. (2008). Tocopherols, Tocotrienols, and γ-Oryzanol Contents in Japonica and Indica Subspecies of Rice (*Oryza sativa* L.) Cultivated in Brazil. Cereal Chem..

[66] Kim N.H., Kwak J., Baik J.Y., Yoon M.-R., Lee J.-S., Yoon S.W., Kim I.-H. (2015). Changes in Lipid Substances in Rice during Grain Development. Phytochemistry.

[67] Lampi A.-M., Nurmi T., Ollilainen V., Piironen V. (2008). Tocopherols and Tocotrienols in Wheat Genotypes in the HEALTHGRAIN Diversity Screen. J. Agric. Food Chem..

[68] Labuschagne M., Mkhatywa N., Johansson E., Wentzel B., van Biljon A. (2017). The Content of Tocols in South African Wheat; Impact on Nutritional Benefits. Foods.

[69] Munne-Bosch S. (2005). The Role of alpha-Tocopherol in Plant Stress Tolerance. J. Plant Physiol..

[70] Fisk I.D., White D.A., Carvalho A., Gray D.A. (2006). Tocopherol—An Intrinsic Component of Sunflower Seed Oil Bodies. J. Am. Oil Chem. Soc..

[71] Siles L., Cela J., Munné-Bosch S. (2013). Vitamin E Analyses in Seeds Reveal a Dominant Presence of Tocotrienols over Tocopherols in the Arecaceae Family. Phytochemistry.

[72] Grams G.W., Blessin C.W., Inglett G.E. (1970). Distribution of Tocopherols within the Corn Kernel. J. Am. Oil Chem. Soc..

[73] Ko S.-N., Kim C.-J., Kim H., Kim C.-T., Chung S.-H., Tae B.-S., Kim I.-H. (2003). Tocol Levels in Milling Fractions of Some Cereal Grains and Soybean. J. Am. Oil Chem. Soc..

[74] Morrison W.R., Coventry A.M., Barnes P.J. (1982). The Distribution of Acyl Lipids and Tocopherols in Flours Millstreams. J. Sci. Food Agric..

[75] Picot A., Atanasova-Pénichon V., Pons S., Marchegay G., Barreau C., Pinson-Gadais L., Roucolle J., Daveau F., Caron D., Richard-Forget F. (2013). Maize Kernel Antioxidants and Their Potential Involvement in Fusarium Ear Rot Resistance. J. Agric. Food Chem..

[76] Falk J., Krahnstöver A., van der Kooij T.A.W., Schlensog M., Krupinska K. (2004). Tocopherol and Tocotrienol Accumulation during Development of Caryopses from Barley (*Hordeum vulgare* L.). Phytochemistry.

[77] Matringe M., Ksas B., Rey P., Havaux M. (2008). Tocotrienols, the Unsaturated Forms of Vitamin E, Can Function as Antioxidants and Lipid Protectors in Tobacco Leaves. Plant Physiol..

[78] Kim S.-E., Lee C.-J., Ji C.Y., Kim H.S., Park S.-U., Lim Y.-H., Park W.S., Ahn M.-J., Bian X., Xie Y. (2019). Transgenic Sweetpotato Plants Overexpressing Tocopherol Cyclase Display Enhanced α-Tocopherol Content and Abiotic Stress Tolerance. Plant Physiol. Biochem..

[79] Spicher L., Almeida J., Gutbrod K., Pipitone R., Dörmann P., Glauser G., Rossi M., Kessler F. (2017). Essential Role for Phytol Kinase and Tocopherol in Tolerance to Combined Light and Temperature Stress in Tomato. J. Exp. Bot..

[80] Cela J., Chang C., Munne-Bosch S. (2011). Accumulation of G- Rather than a-Tocopherol Alters Ethylene Signaling Gene Expression in the Vte4 Mutant of Arabidopsis Thaliana. Plant Cell Physiol..

[81] Maeda H., Song W., Sage T.L., DellaPenna D. (2006). Tocopherols Play a Crucial Role in Low-Temperature Adaptation and Phloem Loading in Arabidopsis. Plant Cell.

[82] Chapman J.M., Muhlemann J.K., Gayomba S.R., Muday G.K. (2019). RBOH-Dependent ROS Synthesis and ROS Scavenging by Plant Specialized Metabolites To Modulate Plant Development and Stress Responses. Chem. Res. Toxicol..

[83] Mène-Saffrané L., Jones A.D., DellaPenna D. (2010). Plastochromanol-8 and Tocopherols Are Essential Lipid-Soluble Antioxidants during Seed Desiccation and Quiescence in Arabidopsis. Proc. Natl. Acad. Sci. USA.

[84] Munné-Bosch S., Weiler E.W., Alegre L., Müller M., Düchting P., Falk J. (2007). A-Tocopherol May Influence Cellular Signaling by Modulating Jasmonic Acid Levels in Plants. Planta.

[85] Fang X., Zhao G., Zhang S., Li Y., Gu H., Li Y., Zhao Q., Qi Y. (2019). Chloroplast-to-Nucleus Signaling Regulates MicroRNA Biogenesis in Arabidopsis. Dev. Cell.

[86] Atkinson N.J., Urwin P.E. (2012). The Interaction of Plant Biotic and Abiotic Stresses: From Genes to the Field. J. Exp. Bot..

[87] Balmer D., Flors V., Glauser G., Mauch-Mani B. (2013). Metabolomics of Cereals under Biotic Stress: Current Knowledge and Techniques. Front. Plant Sci..

[88] Pandey P., Irulappan V., Bagavathiannan M.V., Senthil-Kumar M. (2017). Impact of Combined Abiotic and Biotic Stresses on Plant Growth and Avenues for Crop Improvement by Exploiting Physio-Morphological Traits. Front. Plant Sci..

[89] Perrone G., Ferrara M., Medina A., Pascale M., Magan N. (2020). Toxigenic Fungi and Mycotoxins in a Climate Change Scenario: Ecology, Genomics, Distribution, Prediction and Prevention of the Risk. Microorganisms.

[90] Papendick R., Cook R. (1974). Plant water stress and development of Fusarium foot rot in Wheat Subjected to Different Cultural Practices. Phytopathology.

[91] Wildermuth G.B., Morgan J.M. (2004). Genotypic Differences in Partial Resistance to Crown Rot Caused by Fusarium Pseudograminearum in Relation to an Osmoregulation Gene in Wheat. Australas. Plant Pathol..

[92] Guo B., Chen Z.-Y., Lee R.D., Scully B.T. (2008). Drought Stress and Preharvest Aflatoxin Contamination in Agricultural Commodity: Genetics, Genomics and Proteomics. J. Integr. Plant Biol..

[93] Vaughan M.M., Huffaker A., Schmelz E.A., Dafoe N.J., Christensen S.A., McAuslane H.J., Alborn H.T., Allen L.H., Teal P.E.A. (2016). Interactive Effects of Elevated [CO_2_] and Drought on the Maize Phytochemical Defense Response against Mycotoxigenic Fusarium Verticillioides. PLoS ONE.

[94] Abbas H., Mascagni H., Bruns H., Shier W. (2012). Effect of Planting Density, Irrigation Regimes, and Maize Hybrids with Varying Ear Size on Yield, and Aflatoxin and Fumonisin Contamination Levels. Am. J. Plant Sci..

[95] Desaint H., Aoun N., Deslandes L., Vailleau F., Roux F., Berthomé R. (2021). Fight Hard or Die Trying: When Plants Face Pathogens under Heat Stress. New Phytol..

[96] Ferrigo D., Raiola A., Causin R. (2016). Fusarium Toxins in Cereals: Occurrence, Legislation, Factors Promoting the Appearance and Their Management. Molecules.

[97] Gardiner D.M., Kazan K., Praud S., Torney F.J., Rusu A., Manners J.M. (2010). Early Activation of Wheat Polyamine Biosynthesis during Fusarium Head Blight Implicates Putrescine as an Inducer of Trichothecene Mycotoxin Production. BMC Plant Biol..

[98] Audenaert K., Vanheule A., Höfte M., Haesaert G. (2013). Deoxynivalenol: A Major Player in the Multifaceted Response of Fusarium to Its Environment. Toxins.

[99] Gupta A., Hisano H., Hojo Y., Matsuura T., Ikeda Y., Mori I.C., Senthil-Kumar M. (2017). Global Profiling of Phytohormone Dynamics during Combined Drought and Pathogen Stress in Arabidopsis Thaliana Reveals ABA and JA as Major Regulators. Sci. Rep..

[100] Vellosillo T., Vicente J., Kulasekaran S., Hamberg M., Castresana C. (2010). Emerging Complexity in Reactive Oxygen Species Production and Signaling during the Response of Plants to Pathogens. Plant Physiol..

[101] Baxter A., Mittler R., Suzuki N. (2014). ROS as Key Players in Plant Stress Signalling. J. Exp. Bot..

[102] Waśkiewicz A., Morkunas I., Bednarski W., Mai V.C., Formela M., Beszterda M., Wiśniewska H., Goliński P. (2014). Deoxynivalenol and Oxidative Stress Indicators in Winter Wheat Inoculated with Fusarium Graminearum. Toxins.

[103] Desmond O.J., Manners J.M., Stephens A.E., Maclean D.J., Schenk P.M., Gardiner D.M., Munn A.L., Kazan K. (2008). The Fusarium Mycotoxin Deoxynivalenol Elicits Hydrogen Peroxide Production, Programmed Cell Death and Defence Responses in Wheat. Mol. Plant Pathol..

[104] Pastuszak J., Szczerba A., Dziurka M., Hornyák M., Kopeć P., Szklarczyk M., Płażek A. (2021). Physiological and Biochemical Response to Fusarium Culmorum Infection in Three Durum Wheat Genotypes at Seedling and Full Anthesis Stage. Int. J. Mol. Sci..

[105] Liu H., Wu H., Wang Y., Wang H., Chen S., Yin Z. (2021). Comparative Transcriptome Profiling and Co-Expression Network Analysis Uncover the Key Genes Associated Withearly-Stage Resistance to Aspergillus Flavus in Maize. BMC Plant Biol..

[106] Lambarey H., Moola N., Veenstra A., Murray S., Suhail Rafudeen M. (2020). Transcriptomic Analysis of a Susceptible African Maize Line to Fusarium Verticillioides Infection. Plants.

[107] Kelley R.Y., Williams W.P., Mylroie J.E., Boykin D.L., Harper J.W., Windham G.L., Ankala A., Shan X. (2012). Identification of Maize Genes Associated with Host Plant Resistance or Susceptibility to Aspergillus Flavus Infection and Aflatoxin Accumulation. PLoS ONE.

[108] Rampersad S.N. (2020). Pathogenomics and Management of Fusarium Diseases in Plants. Pathogens.

[109] Montibus M., Pinson-Gadais L., Richard-Forget F., Barreau C., Ponts N. (2015). Coupling of Transcriptional Response to Oxidative Stress and Secondary Metabolism Regulation in Filamentous Fungi. Crit. Rev. Microbiol..

[110] Gil-Serna J., Vázquez C., Patiño B. (2020). Genetic Regulation of Aflatoxin, Ochratoxin A, Trichothecene, and Fumonisin Biosynthesis: A Review. Int. Microbiol. Off. J. Span. Soc. Microbiol..

[111] Szarka A., Tomasskovics B., Bánhegyi G. (2012). The Ascorbate-Glutathione-α-Tocopherol Triad in Abiotic Stress Response. Int. J. Mol. Sci..

[112] Krieger-Liszkay A., Trebst A. (2006). Tocopherol Is the Scavenger of Singlet Oxygen Produced by the Triplet States of Chlorophyll in the PSII Reaction Centre. J. Exp. Bot..

[113] Bari R., Jones J.D.G. (2009). Role of Plant Hormones in Plant Defence Responses. Plant Mol. Biol..

[114] Berens M.L., Berry H.M., Mine A., Argueso C.T., Tsuda K. (2017). Evolution of Hormone Signaling Networks in Plant Defense. Annu. Rev. Phytopathol..

[115] Wang L., Li Q., Liu Z., Surendra A., Pan Y., Li Y., Zaharia L.I., Ouellet T., Fobert P.R. (2018). Integrated Transcriptome and Hormone Profiling Highlight the Role of Multiple Phytohormone Pathways in Wheat Resistance against Fusarium Head Blight. PLoS ONE.

[116] Makandar R., Nalam V.J., Lee H., Trick H.N., Dong Y., Shah J. (2012). Salicylic Acid Regulates Basal Resistance to Fusarium Head Blight in Wheat. Mol. Plant-Microbe Interact..

[117] Li G., Yen Y. (2008). Jasmonate and Ethylene Signaling Pathway May Mediate Fusarium Head Blight Resistance in Wheat. Crop Sci..

[118] Gunnaiah R., Kushalappa A.C., Duggavathi R., Fox S., Somers D.J. (2012). Integrated Metabolo-Proteomic Approach to Decipher the Mechanisms by Which Wheat QTL (Fhb1) Contributes to Resistance against Fusarium Graminearum. PLoS ONE.

[119] Wang Y., Zhou Z., Gao J., Wu Y., Xia Z., Zhang H., Wu J. (2016). The Mechanisms of Maize Resistance to Fusarium Verticillioides by Comprehensive Analysis of RNA-Seq Data. Front. Plant Sci..

[120] Luo M., Brown R.L., Chen Z.-Y., Menkir A., Yu J., Bhatnagar D. (2011). Transcriptional Profiles Uncover Aspergillus Flavus-Induced Resistance in Maize Kernels. Toxins.

[121] Wang Y., Mostafa S., Zeng W., Jin B. (2021). Function and Mechanism of Jasmonic Acid in Plant Responses to Abiotic and Biotic Stresses. Int. J. Mol. Sci..

[122] Yi S.Y., Shirasu K., Moon J.S., Lee S.-G., Kwon S.-Y. (2014). The Activated SA and JA Signaling Pathways Have an Influence on Flg22-Triggered Oxidative Burst and Callose Deposition. PLoS ONE.

[123] Kumaraswamy K.G., Kushalappa A.C., Choo T.M., Dion Y., Rioux S. (2011). Mass Spectrometry Based Metabolomics to Identify Potential Biomarkers for Resistance in Barley against Fusarium Head Blight (*Fusarium graminearum*). J. Chem. Ecol..

[124] Smirnoff N. (2010). Tocochromanols: Rancid Lipids, Seed Longevity, and Beyond. Proc. Natl. Acad. Sci. USA.

[125] Reddanna P., Krishna Rao M., Channa Reddy C. (1985). Inhibition of 5-Lipoxygenase by Vitamin E. FEBS Lett..

[126] Khanna S., Roy S., Ryu H., Bahadduri P., Swaan P.W., Ratan R.R., Sen C.K. (2007). Molecular Basis of Vitamin E Action. Tocotrienol Modulates 12- Lipoxygenase, a Key Mediator of Glutamate-Induced Neurodegeneration. J. Biol. Chem..

[127] Gao X., Brodhagen M., Isakeit T., Brown S.H., Göbel C., Betran J., Feussner I., Keller N.P., Kolomiets M.V. (2009). Inactivation of the Lipoxygenase ZmLOX3 Increases Susceptibility of Maize to *Aspergillus* Spp.. Mol. Plant-Microbe Interact..

[128] Nalam V.J., Alam S., Keereetaweep J., Venables B., Burdan D., Lee H., Trick H.N., Sarowar S., Makandar R., Shah J. (2015). Facilitation of Fusarium Graminearum Infection by 9-Lipoxygenases in Arabidopsis and Wheat. Mol. Plant-Microbe Interact..

[129] Brodhagen M., Tsitsigiannis D.I., Hornung E., Goebel C., Feussner I., Keller N.P. (2008). Reciprocal Oxylipin-Mediated Cross-Talk in the Aspergillus-Seed Pathosystem. Mol. Microbiol..

[130] Scarpari M., Punelli M., Scala V., Zaccaria M., Nobili C., Ludovici M., Camera E., Fabbri A.A., Reverberi M., Fanelli C. (2014). Lipids in Aspergillus Flavus-Maize Interaction. Front. Microbiol..

[131] Qiu M., Wang Y., Sun L., Deng Q., Zhao J. (2021). Fatty Acids and Oxylipins as Antifungal and Anti-Mycotoxin Agents in Food: A Review. Toxins.

[132] Burow G.B., Nesbitt T.C., Dunlap J., Keller N.P. (1997). Seed Lipoxygenase Products Modulate Aspergillus Mycotoxin Biosynthesis. Mol. Plant-Microbe Interact..

[133] Nobili C., D’Angeli S., Altamura M.M., Scala V., Fabbri A.A., Reverberi M., Fanelli C. (2014). ROS and 9-Oxylipins Are Correlated with Deoxynivalenol Accumulation in the Germinating Caryopses of *Triticum aestivum* after *Fusarium graminearum* Infection. Eur. J. Plant Pathol..

[134] Battilani P., Lanubile A., Scala V., Reverberi M., Gregori R., Falavigna C., Dall’asta C., Park Y.-S., Bennett J., Borrego E.J. (2018). Oxylipins from Both Pathogen and Host Antagonize Jasmonic Acid-Mediated Defence via the 9-Lipoxygenase Pathway in *Fusarium Verticillioides* Infection of Maize. Mol. Plant Pathol..

[135] Andrade J.C., Morais-Braga M.F.B., Guedes G.M.M., Tintino S.R., Freitas M.A., Menezes I.R.A., Coutinho H.D.M. (2014). Enhancement of the Antibiotic Activity of Aminoglycosides by Alpha-Tocopherol and Other Cholesterol Derivates. Biomed. Pharmacother..

[136] Ghimire B.K., Seong E.S., Yu C.Y., Kim S.-H., Chung I.-M. (2017). Evaluation of Phenolic Compounds and Antimicrobial Activities in Transgenic Codonopsis Lanceolata Plants via Overexpression of the γ-Tocopherol Methyltransferase (γ-Tmt) Gene. South Afr. J. Bot..

[137] Hajji A., Bnejdi F., Saadoun M., Ben Salem I., Nehdi I., Sbihi H., Alharthi F.A., El Bok S., Boughalleb-M’Hamdi N. (2020). High Reserve in δ-Tocopherol of Peganum Harmala Seeds Oil and Antifungal Activity of Oil against Ten Plant Pathogenic Fungi. Molecules.

[138] Koval D., Plocková M., Kyselka J., Skřivan P., Sluková M., Horáčková Š. (2020). Buckwheat Secondary Metabolites: Potential Antifungal Agents. J. Agric. Food Chem..

[139] Norton R.A. (1999). Inhibition of Aflatoxin B(1) Biosynthesis in Aspergillus Flavus by Anthocyanidins and Related Flavonoids. J. Agric. Food Chem..

[140] Norton R.A. (1997). Effect of Carotenoids on Aflatoxin B(1) Synthesis by Aspergillus Flavus. Phytopathology.

