# Supplementary material for: Role of Tocochromanols in Tolerance of Cereals to Biotic Stresses: Specific Focus on Pathogenic and Toxigenic Fungal Species
Source: Int J Mol Sci. 2022 Aug 18;23(16):9303. doi: 10.3390/ijms23169303 (PMC9408828; doi:10.3390/ijms23169303)
Supplement: Supplementary file 1 [file ijms-23-09303-s001.zip › ijms-1826541-supplementary.pdf]

Table S1: Tocochromanol composition of major cereal crops

	α-T	β-T	γ-T	δ-T	α-T3	β-T3	γ-T3	δ-T3	Ref.
Maize	1.43 - 32.54	ND	2.37 - 63.28	0.20 - 4.27	ND	ND	ND	ND	[56]
Maize	9.79	0.47	8.32	0.79	3.83	ND	17.11	0.81	[58]
Maize	2.43	ND	31.23	ND	1.73	ND	ND	ND	[60]
Maize	8.62	0.55	11.57	6.33	2.41	0.39	6.07	0.81	[59]
Average maize (%)	13.85 (26)	0.51 (1)	17.57 (33)	6.14 (11)	3.12 (6)	0.39 (1)	11.59 (21)	0.81 (1)	
Oat	84.40	10.20	0.30	0.10	60±	5.90	0.30	ND	[38]
Oat	5.40	ND	3.20	traces	4.20	traces	2.10	0.90	[61]
Oat	0.85	0.78	ND	ND	2.68	10.80	ND	ND	[62]
Average oat (%)	30.22 (45)	3.66 (6)	1.17 (2)	0.03 (<0.5)	22.29 (33)	8.35 (13)	0.8 (1)	0.3 (<0.5)	
Rice	0.4 - 3.33	ND - 0.2	1.7 - 5.14	ND - 0.35	0.5 - 3.1	ND	ND - 2.23	ND - 0.62	[61]
Rice Unpigmented	16.61	2.08	8.23	1.46	6.46	1.46	21.94	1.61	[57]
Rice Pigmented	10.14	0.92	5.10	0.91	6.60	0.11	27.88	1.46	[57]
Ilpum rice	5.46	0.34	0.46	0.01	3.30	ND	3.25	0.32	[66]
Dasan rice	1.42	0.09	1.20	0.07	0.45	ND	10.34	0.37	[66]
Rice	6.43 - 12.67	ND	1.39 - 4.15	0.86 - 1.52	2.32 - 10.01	ND	12.88 - 32.75	0.96 - 2.01	[63]
Rice Japonica	10.00	ND	1.40	ND	7.00	ND	5.80	ND	[65]
Rice Indica	4.80	ND	1.30	ND	2.30	ND	7.80	ND	[65]
Average rice (%)	13.35 (26)	0.39 (1)	6.26 (10)	0.42 (2)	15.73 (15)	0.17 (<0.5)	21.83 (43)	0.61 (2)	
Barley	0.78	traces	traces	traces	0.59	traces	traces	traces	[61]
Barley	10.31	ND	2.47	0.08	13.92	ND	4.57	0.18	[52]
Barley	3.84	ND	ND	ND	17.09	1.32	4.73	ND	[61]
Average barley (%)	4.98 (22)	ND	1.24 (6)	0.04 (<0.5)	10.53 (48)	0.66 (3)	4.65 (21)	0.09 (<0.5)	
Wheat	15.40	5.00	ND	ND	5.00	19.60	ND	ND	[61]
Spring wheat	11.93	4.51	ND	ND	2.43	12.20	ND	ND	[52]
Winter wheat	10.58	4.01	ND	ND	1.86	9.82	ND	ND	[52]
Wheat	13.00	6.00	ND	ND	5.00	25.40	ND	ND	[67]
Wheat	13.30	6.43	ND	ND	4.93	24.00	ND	0.60	[68]
Wheat	12.70	4.73	ND	ND	13.00	38.23	ND	ND	[54]
Wheat	6.06	4.23	ND	ND	1.05	23.68	ND	ND	[61]
Average wheat (%)	12.02 (27)	5.30 (12)	ND	ND	5.09 (11)	22.55 (50)	ND	0.08 (<0.5)	
